# *ITGB5* Plays a Key Role in *Escherichia coli* F4ac-Induced Diarrhea in Piglets

**DOI:** 10.3389/fimmu.2019.02834

**Published:** 2019-12-11

**Authors:** Wenwen Wang, Yang Liu, Hui Tang, Ying Yu, Qin Zhang

**Affiliations:** ^1^Shandong Provincial Key Laboratory of Animal Biotechnology and Disease Control and Prevention, Shandong Agricultural University, Tai'an, China; ^2^College of Animal Science and Technology, Nanjing Agricultural University, Nanjing, China; ^3^Key Laboratory of Animal Genetics, Breeding and Reproduction, Ministry of Agriculture, College of Animal Science and Technology, China Agricultural University, Beijing, China

**Keywords:** pig, enterotoxigenic *Escherichia coli*, diarrhea, proteomics, CRISPR/Cas9

## Abstract

Enterotoxigenic *Escherichia coli* (ETEC) that expresses F4ac fimbriae is the major pathogenic microorganism responsible for bacterial diarrhea in neonatal piglets. The susceptibility of piglets to ETEC F4ac is determined by a specific receptor on the small intestinal epithelium surface. We performed an iTRAQ-labeled quantitative proteome analysis using a case-control design in which susceptible and resistant full-sib piglets were compared for the protein expression levels. Two thousand two hundred forty-nine proteins were identified, of which 245 were differentially expressed (fold change > 1.5, FDR-adjusted *P* < 0.05). The differentially expressed proteins fell into four functional classes: (I) cellular adhesion and binding, (II) metabolic process, (III) apoptosis and proliferation, and (IV) immune response. The integrin signaling pathway merited particular interest based on a pathway analysis using statistical overexpression and enrichment tests. Genomic locations of the integrin family genes were determined based on the most recent porcine genome sequence assembly (Sscrofa11.1). Only one gene, *ITGB5*, which encodes the integrin β5 subunit that assorts with the αv subunit to generate integrin αvβ5, was located within the SSC13q41 region between 13:133161078 and 13:139609422, where strong associations of markers with the ETEC F4ac susceptibility were found in our previous GWAS results. To identify whether integrin αvβ5 is the ETEC F4acR, we established an experimental model for bacterial adhesion using IPEC-J2 cells. Then, the *ITGB5* gene was knocked out in IPEC-J2 cell lines using CRISPR/Cas9, resulting in a biallelic deletion cell line (*ITGB5*^−/−^). Disruption of *ITGB5* significantly reduced ETEC F4ac adhesion to porcine intestinal epithelial cells. In contrast, overexpression of *ITGB5* significantly enhanced the adhesion. A GST pull-down assay with purified FaeG and ITGB5 also showed that FaeG binds directly to ITGB5. Together, the results suggested that *ITGB5* is a key factor affecting the susceptibility of piglets to ETEC F4ac.

## Introduction

Enterotoxigenic *Escherichia coli* (ETEC)-induced diarrhea is one of the major diseases in neonatal and weaned piglets, resulting in severe economic losses in the swine industry. Among the five different fimbriae isolated from diarrheic pigs, F4 (K88) is the most prevalent (1). Three antigenically distinct subgroups (F4ab, F4ac, and F4ad) have been identified in F4 fimbriae, of which the F4ac variant is the most common (2, 3). Sellwood et al. (4) first proposed the “specific K88 receptor” hypothesis, which states that the susceptibility of piglets to ETEC F4 is determined by the presence or absence of a specific F4 receptor on the small intestinal epithelium surface of the animal.

The gene encoding the F4ac receptor (F4acR) has been mapped to the SSC13q41 region in two linkage studies (5, 6). Subsequently, it was refined to a 5.7 cm interval by using a meta-analysis (7), and it was further narrowed down to a 1.6 cm interval by using a pedigree disequilibrium test (PDT) (8). Within this interval, we identified 18 SNPs through a genome-wide association study (GWAS), and these were strongly associated with the susceptibility of piglets to ETEC F4ac (9), and *HEG1* and *ITGB5* emerged as the most promising candidate gene for F4acR. Although some further studies have been carried out to reveal the molecular basis of the susceptibility of piglets to ETEC F4ac (10, 11), the role of the F4acR protein and its encoding gene remain uncertain.

Because post-transcriptional and translational regulatory mechanisms affect protein levels in eukaryotes, mRNA abundance could be a misleading indicator of protein levels (12). In contrast, proteomics more directly measures protein levels and may provide a better view into the molecular basis of ETEC F4ac susceptibility. Using iTRAQ (isobaric tag for relative and absolute quantitation) or other labeling methods, it is possible to quantitatively compare the protein levels of up to eight samples in a single mass spectrometry experiment (13). We therefore conducted a high-throughput proteomics analysis to compare protein expression in ETEC F4ac-susceptible and resistant piglets, focusing primarily on identifying the potential F4acR protein(s), and the corresponding gene(s). Four pairs of full-sib piglets, each consisting of one susceptible and one resistant to ETEC F4ac, were analyzed. The eight samples were multiplexed using iTRAQ and subjected to LC (liquid chromatography)–MS/MS (tandem mass spectrometry) to identify differentially expressed proteins (DEPs). Among the DEPs detected, integrin αvβ5 was considered as a potential F4acR protein. *ITGB5*, which encodes integrin subunit beta 5, was disrupted using methods based on CRISPR/Cas9. Cells containing the *ITGB5* knockout, and cells in which *ITGB5* was overexpressed, were tested for their ability to adhere to ETEC F4ac. The results provided direct evidence for the role of *ITGB5* in infection by ETEC F4ac and helped to clarify the mechanisms underlying piglet susceptibility to diarrhea.

## Results

### Adhesion Phenotypes

One hundred eighty-nine Large White piglets were examined for the adhesion phenotype by co-culturing epithelial cells from their jejunums with ETEC F4ac. A total of 83 piglets were found to be adhesive, 14 weakly adhesive, and 92 were non-adhesive. Four pairs of full-sibs, each with one adhesive, and one non-adhesive piglet, were selected for proteomics analysis.

### iTRAQ Profiling of Adhesive vs. Non-adhesive Samples

Protein samples from the four pairs of full-sibs were labeled with isobaric tags (pair 1, 113:117; pair 2, 114:118; pair 3, 115:119; and pair 4, 116:121) and then subjected to quantitative proteomics analysis. After combining data from the four pairs, we identified 17,155 unique peptides from 43,261 spectra, corresponding to 2,249 proteins (a 1% FDR threshold was imposed for both peptides and proteins). Sample quality were inferred from the wide range of protein classes detected in the analysis. Using the PANTHER classification system, the 2,249 identified proteins fell into 29 families (Figure S1).

A protein was defined as differentially expressed protein (DEP) when its fold-change (FC) of expression between adhesive and one non-adhesive samples was >1.5 at an FDR-adjusted significant level of *P* < 0.05 (Figure S2). A total of 245 DEPs were identified, of which 117 (47.8%) were more abundant in adhesive samples, and 128 (52.2%) were less abundant (Tables S1, S2).

### Protein–Protein Interaction Network

**Figure 1:**
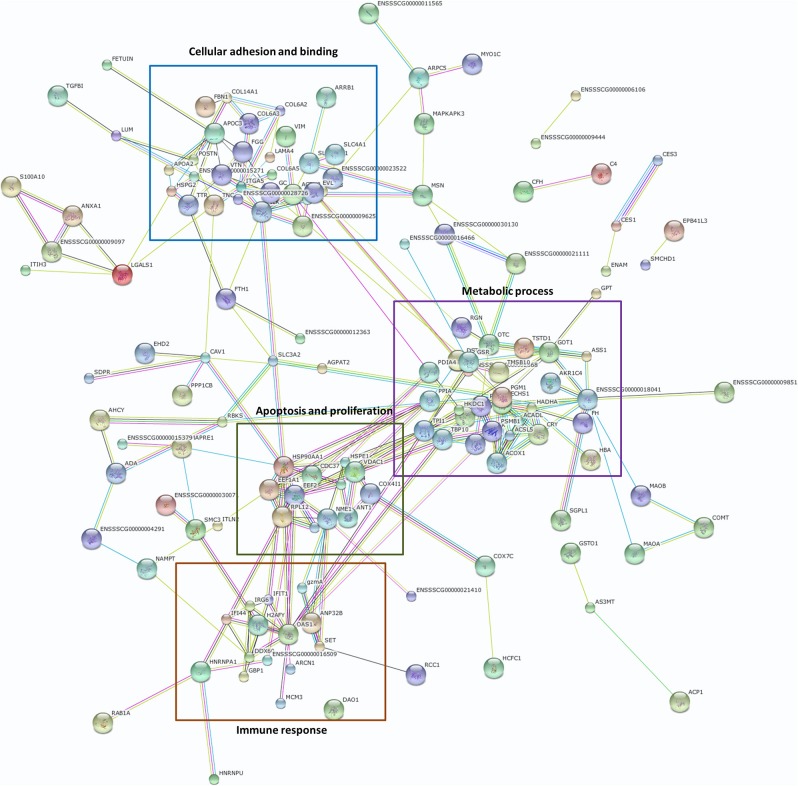
Protein–protein interactions based on a search of the STRING database version 10.5 (https://string-db.org/). DEPs formed a tightly interconnected network. The four boxed regions are described in the text.

To identify possible functions associated with the differentially expressed proteins, we constructed a protein–protein interaction network using the DEPs as seed nodes (Figure 1). Four sub-clusters were apparent. The first sub-cluster is associated with cellular adhesion and binding, and includes adhesion proteins such as ITGA5, COL6A3, ACTN2, CAV1, ILK, COL14A1, and VTN. Since the susceptibility of piglets to ETEC F4ac is determined by the presence of F4acR on the surface of the small intestinal epithelium, these proteins are potentially involved in the diarrhea induced by ETEC F4ac. The other three sub-clusters are associated with metabolic processes, apoptosis and proliferation, and the immune response. Members of these groups have been identified by mRNA expression profiling of porcine epithelial cells infected with ETEC F4ac (10).

### Pathway Analysis of the Genes Corresponding to DEPs

**Figure 2:**
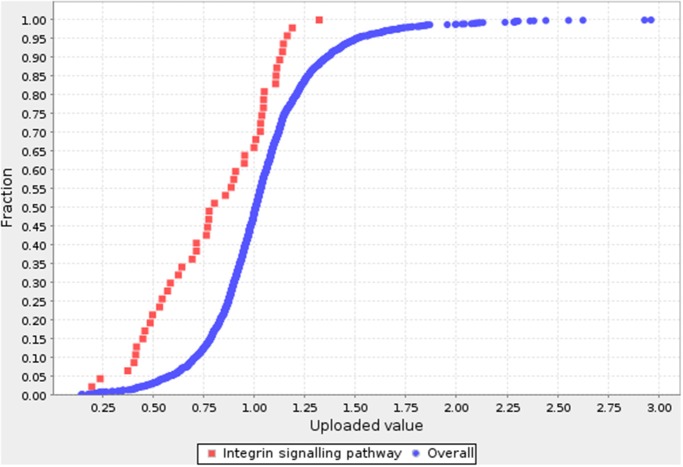
Pathway analysis for DEPs. The blue curve is the overall distribution for all proteins. The red curve is the integrin signaling pathway. Fold-change is shown on the X axis, and cumulative fraction is shown on the Y axis.

A pathway enrichment analysis was conducted to gain deeper insight into the functions of the differentially expressed proteins. The functions were assessed using the statistical overrepresentation and statistical enrichment tests (14). The statistical overrepresentation test is based conceptually on the simple binomial test (15) to determine whether a particular pathway of genes is overrepresented or underrepresented. The statistical enrichment test uses the Mann–Whitney test (16) to determine whether any pathway has numeric values that are non-randomly distributed with respect to the entire list of values. Of note was that only the integrin signaling pathway was significantly enriched (*P* < 0.05) by either of the two tests. Figure 2 compares the distributions of the proteins from the integrin signaling pathway and the reference proteins. The blue curve is the overall distribution for all proteins and the one is the integrin signaling pathway.

### Chromosomal Locations of the Integrin Family Genes

The results of the protein–protein interaction network analysis and the KEGG analysis of the DEPs suggest that the protein(s) responsible for the adhesion of ETEC F4ac to the small intestinal epithelium surface of piglets are very likely member(s) of the integrin family. We therefore focused on integrin family proteins in the subsequent analysis.

**Figure 3:**
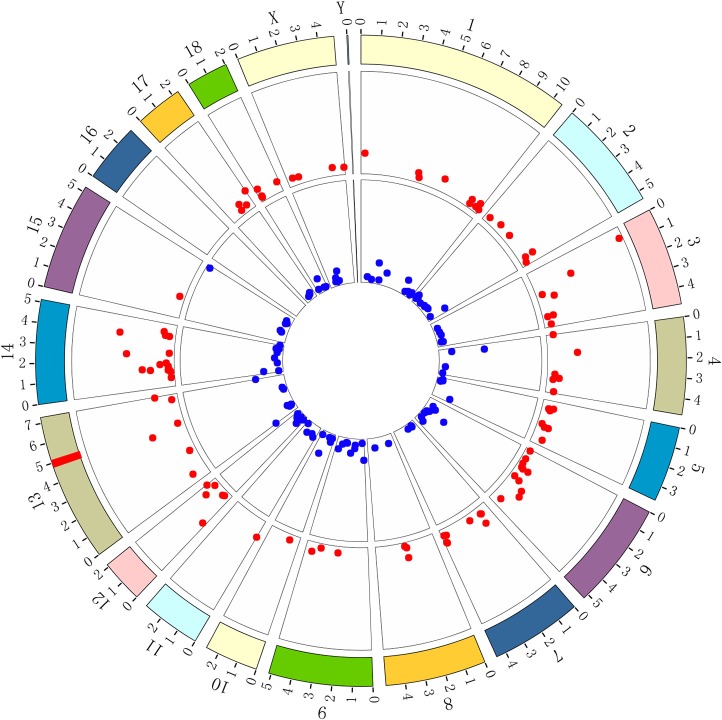
BioCircos was used to visualize the locations of DEG loci on chromosomes. Red points represent genes expressed at higher levels in adhesive piglets; blue points represent genes expressed at lower levels in adhesive piglets. The red line in the band at 13q41 locates the locus that encodes ETEC F4acR based on previous studies. The distance from location to outer periphery is –log (*p*-value).

It has been commonly accepted that the gene(s) encoding ETEC F4acR are located in the SSC13q41 region (2, 5, 6, 8, 9, 17, 18). We used BioCircos to visualize the chromosomal locations of the genes corresponding to the differentially expressed proteins. As shown in Figure 3, these genes are found on all chromosomes except SSC16. BioMart was used to assign chromosomal locations for genes of the integrin family (Table 1). Only one gene, *ITGB5*, is located in the SSC13q41 region.

**Table 1:** Integrin family gene loci.

**Associated gene name**	**Chromosome**	**Gene start (bp)^a^**	**Gene end (bp)**
*ITGA1*	16	32185484	32295491
*ITGA2*	16	32336414	32433371
*ITGA2B*	12	18776205	18877382
*ITGA3*	12	26235324	26271561
*ITGA4*	15	86946996	87029729
*ITGA5*	5	19583451	19611209
*ITGA6*	15	78504312	78589645
*ITGA7*	5	21127015	21147909
*ITGA8*	10	46132741	46303652
*ITGA9*	13	22298498	22651675
*ITGA10*	4	99414915	99442466
*ITGA11*	1	166173135	166310972
*ITGAD*	3	17136161	17169776
*ITGAE*	12	49810054	49899055
*ITGAL*	3	17817568	17858086
*ITGAM*	3	17134864	17265533
*ITGAV*	15	91604661	91711841
*ITGAX*	3	17178725	17201388
*ITGB1*	10	56078401	56173795
*ITGB1BP1*	3	126905810	126922045
*ITGB1BP2*	X	57316490	57321607
*ITGB2*	13	207510960	207544146
*ITGB3*	12	16693514	16752292
*ITGB4*	12	5651021	5675543
*ITGB5*	13	135467337	135590352
*ITGB6*	15	67041519	67175254
*ITGB7*	5	18417198	18434526
*ITGB8*	9	89341141	89450696
*ITGBL1*	11	70041968	70248362
*ITFG2*	5	67290112	67305758

a *Derived from the most recent porcine genome sequence assembly (Sscrofa11.1)*.

### CRISPR/Cas9-Mediated *ITGB5* Gene Deficiency

**Figure 4:**
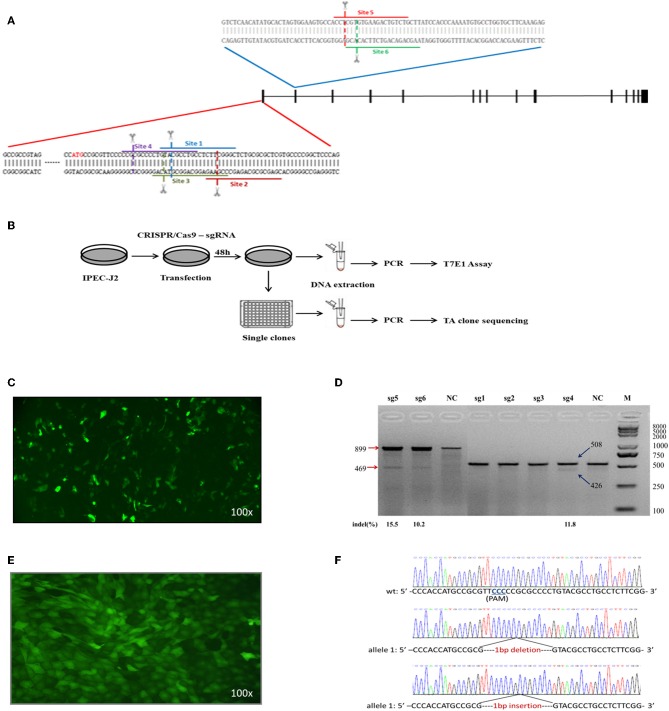
Construction and identification of the cell line containing biallelic mutation (*ITGB5*^−/−^). **(A)** *ITGB5* target site position. sgRNAs for six sites were designed based on the sequence at exons 1 and 2. **(B)** Protocol for gene disruption. The CRISPR/Cas9–sgRNA target vector was transfected into IPEC-J2 cells, and cellular DNA was collected for PCR analysis 48 h after transfection. The most efficient target vector was used for gene knockout. Cells collected 48 h after transfection were inoculated into 96-well plates. G418 selection was used to obtain single clones. DNA collected from single clones was sequenced. **(C)** The CRISPR/Cas9–sgRNA knockout vector and pEGFP-C1 plasmids were transfected into IPEC-J2 cells. **(D)** Cleavage efficiency of CRISPR/Cas9–sgRNA at six target sites was quantified with the T7EN1-cleavage assay and analyzed using ImageJ. **(E)** Image of IPEC-J2-sg4-6 cell line. **(F)** Sequencing results from targeted regions in the IPEC-J2-sg4-6 cell line.

Six single-guide RNAs (sgRNA1 to sgRNA6) were designed to target sites within exon 1 and exon 2 of the *ITGB5* coding sequence (Figure 4A). The workflow to establish an *ITGB5* gene knockout cell line is summarized in Figure 4B. To test the luciferase signal, pEGFP-C1 plasmids, which included genes encoding enhanced green fluorescent protein (eGFP), were co-transfected with CRISPR/Cas9–sgRNA into IPEC-J2 cells to confirm DNA uptake (Figure 4C). T7 endonuclease I (T7EN1)-cleavage assays were used to detect gene targeting efficiency. As shown in Figure 4D, sgRNA1, sgRNA2, and sgRNA3 did not generate any significant cleavage, whereas sgRNA4, sgRNA5, and sgRNA6 exhibited cleavage efficiencies of 11.8, 10.2, and 15.5%, respectively. As the sgRNA4 target site is located in exon1, we used sgRNA4 in subsequent experiments.

The minimal lethal dose of puromycin was determined to be 600 μg/mL for IPEC-J2 and was used to obtain 21 cell lines. Green fluorescence was detected in all cell lines by fluorescence microscopy (Figure 4E). One cell line (IPEC-J2-sg4-6) contained a compound heterozygous knockout (*ITGB5*^−/−^) in which one allele was a 1-nucleotide deletion (based on sequencing 29 TA clones) and the other allele was a 1-nucleotide insertion (based on sequencing 21 TA clones) in exon 1 of *ITGB5* (Figure 4F). IPEC-J2-sg4-6 was therefore used to assess the function of *ITGB5*.

### Effects of Knockout and Overexpression of *ITGB5* on ETEC F4ac Adhesion to IPEC-J2 Cells

**Figure 5:**
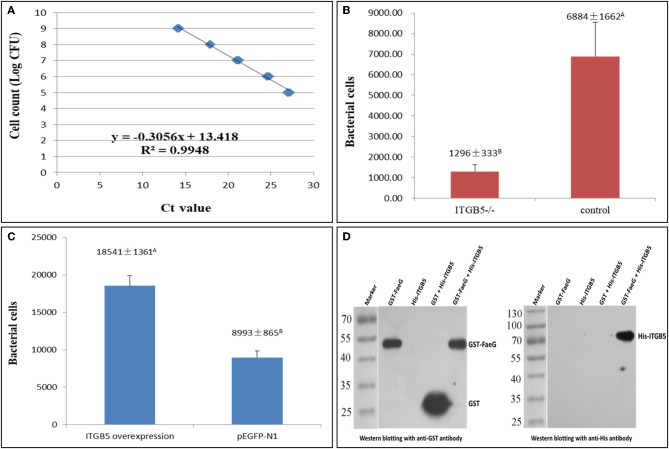
Role of *ITGB5* gene on ETEC F4ac adhesion to IPEC-J2 cells. **(A)** Standard curve for quantification of bacterial adherence to IPEC-J2 cells. **(B)** Reduction of ETEC F4ac adhesion after *ITGB5* knockout in IPEC-J2. **(C)** Bacterial adhesion after *ITGB5* overexpression in IPEC-J2 cells. **(D)** GST pull-down assays. The binding between the recombinant FaeG and ITGB5 proteins was studied using the Pierce™ GST Protein Interaction Pull-Down Kit. Western blotting with anti-GST and anti-His antibody was used for detection. Data are shown as means ± SD; *n* = 3. In each group, values without a common letter were significantly different (*P* < 0.01).

To quantify ETEC F4ac adherence to IPEC-J2 cells, a standard curve (Figure 5A) was prepared using a range of bacterial concentrations (1 × 10^5^-1 × 10^9^ CFU/mL). Bacterial adhesion to IPEC-J2 cells was evaluated by real-time PCR. *ITGB5*^−/−^ cells showed significantly less adherence in comparison to cells transfected with an empty vector (Figure 5B). Overexpression of *ITGB5* in IPEC-J2 cells resulted in a significant increase in mRNA expression (*P* < 0.01) and increased ETEC F4ac adherence to porcine intestinal epithelial cells (*P* < 0.01; Figure 5C).

### Verification of the Interaction Between ITGB5 and FaeG

Previous studies have demonstrated that the fimbrial subunit FaeG is the most prominent part for F4 adherence and is directly involved in the binding of the F4 fimbriae to the host cells (19). To further verify the interaction between FaeG and ITGB5, a GST pull-down assay was conducted. A pull-down assay is an *in vitro* technique used to detect physical interactions between two or more proteins, and it is also an invaluable tool for confirming a predicted protein–protein interaction (20). To increase the solubility of the protein when expressed in prokaryotic cells, we eliminated the transmembrane region of ITGB5, and then the His-ITGB5 and GST-FaeG fusion proteins were expressed in *Escherichia coli* strain Rosetta and purified. GST pull-down results with purified ITGB5 and FaeG demonstrated that ITGB5 binds directly to FaeG *in vitro* (Figure 5D).

## Discussion

The initial step in infection for the ETEC F4ac is to adhere to host enterocytes through fimbriae-mediated recognition of receptors on the host cell surface (11). Sellwood first reported that piglets lacking the appropriate receptors in the intestinal mucosa were resistant to the F4ac infection (4). Identifying the ETEC F4acR protein(s) in piglets is an important step in the efforts to combat enterotoxigenic *Escherichia coli*-associated diarrhea. Erickson et al. (21) and Billey et al. (22) described that F4ac and F4ab bind to two intestinal mucin-type sialoglycoproteins (IMTGP-1 and IMTGP-2) with a molecular mass of 210 and 240 kDa, and that the intestinal transferrin (GP74) with a molecular mass of 74 kDa was shown to be a F4ab-specific receptor (23). Furthermore, Melkebeek et al. (24) identified aminopeptidase N (APN) as an newly discovered receptor for F4ac fimbria, which is involved in oral immune response and clathrin-mediated endocytosis of F4ac fimbriae. Also, many studies were seeking to unravel the gene encoding the F4ac receptor protein. Edfors-Lilja et al. (25) first mapped the F4acR gene to the SSCq41 region, 7.4-cM away from the *TF* locus. Subsequent studies further mapped it between *Sw207–S0075* within SSCq41 (5, 7). Within this region, our group restricted the F4acR gene to a 1.6-cM interval between *S0283* and *SW1876* (10). Further genome-wide association mapping with the Illumina PorcineSNP60 BeadChip revealed that 18 SNPs located between 13:133161078 and 13:139609422 were strongly associated with susceptibility to ETEC F4ac (9).

Despite the current knowledge of ETEC F4ac receptors, there are problems that remain unsolved: it is difficult to locate the exact region of the receptor gene on chromosome 13 and choose the appropriate candidate genes to study, and it is hard to determine which key factors affect the adhesion of ETEC F4ac. The lack of convincing evidence regarding the F4ac receptors and their function motivates further research. In this study, we used an iTRAQ-labeled proteome analysis and a full-sib pair case-control design to identify differentially expressed proteins (DEPs) between F4ac-susceptible and resistant piglets, and we also used it to reveal proteins that are likely to be responsible for the susceptibility of piglets to F4ac.

A total of 245 DEPs were identified, of which 117 (47.8%) were more abundant in cells characterized as adhesive, and 128 (52.2%) were more abundant in those classified as non-adhesive. Analysis of the protein–protein interaction network constructed using the DEPs revealed that they were significantly enriched in functions of (I) cellular adhesion and binding, (II) metabolic processes, (III) apoptosis and proliferation, and (IV) the immune response (Figure 1). Overrepresentation and enrichment tests were used to analyze pathways containing DEPs. After Bonferroni correction, only the integrin signaling pathway was identified by either of the two tests. Since the diarrhea caused by ETEC F4ac infection is thought to be due to the adhesion of the bacteria to the enterocyte brush borders (7), we focused on integrin signaling pathway molecules as interesting candidate proteins.

Integrins are cell surface receptors that participate in cell–cell and extracellular matrix (ECM)-cell interactions (26, 27), and they can also be targeted by pathogenic bacteria, fungi, and viruses. Several human pathogens invade their hosts by taking advantage of integrin-mediated signaling (28). Some pathogenic bacteria, such as *Yersinia enterocolitica, Y. pseudotuberculosis, Helicobacter pylori*, and *Neisseria gonorrhoeae*, can bind integrin receptors directly using specific adhesins (29–31). However, most microorganisms bind integrin indirectly, i.e., they first bind the ECM-binding proteins, and then the integrin receptors bind the arginine-glycine-aspartate motif, such as fibronectin (Fn) and vitronection (Vn), in the ECM proteins (32, 33). The integrin “adhesome network” is estimated to include more than 180 potential signaling and adaptor proteins (34).

Several integrin signaling pathway-related proteins, including integrin alpha-5 and vitronectin (Vn), which were enriched in cellular adhesion and were binding in our analyses, were more abundant in adhesive samples (Table S1). Integrin alpha-5, encoded by *ITGA5*, is a member of the integrin family and functions in cell-surface adhesion and signaling (35). Vitronectin, encoded by *VTN*, is recognized by some integrins and plays a key role in cell-to-substrate adhesion (36).

Integrins are glycoproteins that are generally composed of one α and one β subunit. In mammals, there are 8 different β subunits and 18 different α subunits that can assort with each other to form 24 different integrins with different ligand-binding specificities (26). As mentioned above, many studies have revealed that the gene(s) encoding ETEC F4acR are located in the SSC13q41 region (2, 5, 6, 8, 9, 17, 18). Our previous GWAS study identified 18 SNPs associated with susceptibility to ETEC F4ac, located within the interval from 13:133161078 to 13:139609422 (Table S3) (9). We mapped the integrin family genes onto the most recent porcine genome sequence assembly (Sscrofa11.1) (Table 1) and found only *ITGB5* within SSC13q41, between 13:133161078 and 13:139609422. *ITGB5* encodes the integrin β5 subunit, which combines with the αv subunit to generate integrin αvβ5, a complex that functions in the innate defense system against bacteria (37). Integrin αvβ5 is a major endocytic receptor for vitronectin (Vn) (38–40). Because vitronectin binds both pathogens and epithelial cells, it probably functions as an adapter molecule between them (41). When Vn binds to *Escherichia coli, Staph. aureus, S. pneumoniae, Streptococcous spp*., and *Pseudomonas fluorescens*, it enables more efficient adhesion of the bacteria to epithelial cells (28, 42, 43). In addition, our iTRAQ-labeled proteome analysis showed that Vn was more abundant in adhesive samples (Table S1).

Fimbria act as lectins, which bind to receptors, and destroying receptors completely abolishes the binding of F4ac fimbriae to enterocytes. To test the hypothesis that the ETEC F4acR protein is integrin αvβ5, we generated cell lines in which *ITGB5* was either inactivated by a CRISPR/Cas9-mediated knockout or overexpressed. Both *ITGB5* alleles in the resulting monoclonal cell line IPEC-J2-sg4-6 contained mutations (*ITGB5*^−/−^). As expected, IPEC-J2-sg4-6 (*ITGB5*^−/−^) cells bound significantly less bacteria in an adhesion assay (Figure 5B). In the complementary experiment, overexpression of *ITGB5* in IPEC-J2 cells increased significantly ETEC F4ac adhesion (Figure 5C). The fimbrial subunit FaeG is the most prominent part for F4ac adherence and is directly involved in the binding of the F4ac fimbriae to the receptors (19). Results from GST pull-down assay with purified FaeG and ITGB5 also showed that FaeG binds directly to ITGB5 (Figure 5D). Together, these data suggest that *ITGB5* is a key factor affecting ETEC F4ac susceptibility in Large White piglets. The genetic mechanism of the susceptibility of piglets to ETEC F4ac might not be completely the same over breeds, and more research is required to validate the findings in other breeds.

## Conclusion

In this study, an iTRAQ-labeled quantitative proteome analysis using a case-control design was performed. *ITGB5* was considered to be a promising candidate gene for ETEC F4ac susceptibility in piglets. To test this hypothesis, we established an experimental model for bacterial adhesion using IPEC-J2 cells. *ITGB5* gene knockout significantly reduced ETEC F4ac adhesion to porcine intestinal epithelial cells, and overexpression of *ITGB5* significantly enhanced adhesion. A GST pull-down assay with purified FaeG and ITGB5 also showed that FaeG binds directly to ITGB5. Together, the results suggest that *ITGB5* is a key factor affecting ETEC F4ac susceptibility in Large White piglets.

## Materials and Methods

### Ethics Statement

Animal experiments were carried out in accordance with the Guidelines for Experimental Animals established by the Ministry of Science and Technology (Beijing, China), and all efforts were made to minimize suffering. The protocol was approved by the Institutional Animal Care and Use Ethics Committee of Shandong Agricultural University.

### Materials

A total of 189 Large White piglets, the offspring of seven boars and 31 sows, were used in this study. They were raised under standard indoor conditions at the experimental farm of the Institute of Animal Sciences, Chinese Academy of Agricultural Sciences. ETEC F4 strain 200 (F4ac, C83907, O149:K91) was provided by the China Institute of Veterinary Drug Control, Beijing, China.

### Measurement of Phenotypes

**Figure 6:**
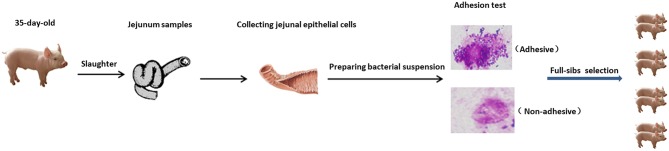
Overview of experimental design for measurement of phenotypes. Four pairs of full-sibs were selected according to their adhesion phenotypes.

The experimental design used to test the susceptibility of piglet intestinal epithelial cells to ETEC F4ac is outlined in Figure 6. The 189 piglets were slaughtered at 35 days of age, and jejunum samples were collected. A 10 cm segment was taken from each of the samples, and the remainder was frozen immediately in liquid nitrogen for later use. The longitudinal axis of the jejunum was cut, and the material was cleaned with a cold hypotonic EDTA solution (5 mmol/L EDTA, pH 7.4). Epithelial cells were obtained by scraping the mucosal surface of the tissue with a glass microscope slide. Using the cells, the piglets were then classified with respect to adhesion phenotype. The *E. coli* strains were cultured, harvested by centrifugation, and resuspended in PBS (pH 7.4) at an optical density of ~1.0 at 520 nm. The cell suspension and the bacterial suspension (0.1 mL each) were mixed in 0.4 mg/mL mannose and incubated for 30 min at room temperature. A drop of the mixture was assessed for bacterial adhesion using a phase contrast microscope. Adhesion phenotypes were classified (adhesive, weakly adhesive, and non-adhesive) in the same way as described previously (44).

To minimize the influence of differences in genetic background and environment between individuals on protein expression, we adapted a full-sib paired case-control design for proteomics analysis, in which four pairs of full-sibs (each with one negative and one positive piglet) from different boars were selected from the 189 piglets.

### Protein Extraction and Quantitation

Samples of intestinal tissues of the eight piglets were ground to a powder in liquid nitrogen using a mortar and pestle. An amount of 200 μL lysis buffer (7 M urea, 2 M thiourea, and 0.1% CHAPS) was added with phenylmethanesulfonyl fluoride (PMSF) and ethylene diamine tetra-acetic acid (EDTA) at final concentrations of 1 and 2 mM, respectively. The suspension was sonicated for 60 s (periods of 0.2 s at 22% amplitude at 2 s intervals). The homogenate was incubated at room temperature for 30 min and then centrifuged at 4 °C and 15,000 × g for 20 min. The supernatant was collected, and the protein concentration was determined using the Bio-Rad Protein assay reagent (Bio-Rad Laboratories, CA, USA).

### Protein Digestion and iTRAQ Labeling

Protein digestion was conducted using a published protocol with minor modifications (45). Briefly, 200 μg of protein from each sample was combined with 10 mM dithiothreitol (DTT) and incubated at 37°C for 1 h. Subsequently, cysteines were blocked by the addition of 40 mM iodoacetamide for 1 h at room temperature in the dark. The supernatant was mixed well with chilled acetone (1:5, v/v) for 2 h at −20°C to precipitate proteins. The protein was diluted 1:3 with 50 mM triethylammonium bicarbonate (TEAB, Applied Biosystems, Milan, Italy) and then incubated with 4 μg trypsin (Promega) at 37°C overnight. The digested peptides were desalted using Sep-Pak C18 cartridges (Waters) and dried in a SpeedVac (Eppendorf).

Desalted peptides were labeled with iTRAQ reagents (Applied Biosystems, Foster City, CA) according to the manufacturer's instructions. The control samples (proteins extracted from piglets phenotyped as non-adhesive) were labeled using iTRAQ labels 117, 118, 119, and 121, and the corresponding case samples (adhesive) were labeled using labels 113–116.

### LC-MS/MS Analysis

First dimension peptide separation was performed with an Ultimate 3000 liquid chromatography system (RIGOL L-3000, Beijing, China) connected to a strong cation exchange (SCX) column. Then, 60 μL of labeled peptides were injected using the microliter-pickup injection mode into a 4.6 × 250 mm SCX column (Agela Durashell C18) that contained 5 μm particles. SCX buffer A was 98% ddH_2_O (adjusted to pH 10 using ammonia) and 2% CAN, and buffer B was 2% ddH_2_O (adjusted to pH 10 using ammonia) and 98% CAN. The flow rate was 0.7 mL/min. Absorbance at 214 nm was measured to monitor elution. From this, 48 fractions were obtained (90 s each) using step gradients of mobile phase B as follows: 5–8% for 5 min, 8–18% for 30 min, 18–32% for 27 min, 32–95% for 2 min and then maintained for 4 min, and decreased to 5% for the final 4 min. The 48 fractions were combined into 10 fractions before second-dimension reverse phase (RP) chromatography. Each fraction was trapped and desalted on an Acclaim PepMap100 precolumn (20 mm × 100 μm, C18, 5 μm) and eluted on an EASY-Spray column (120 mm × 75 μm, C18, 3 μm) for analytical separations. For second-dimensional separation, mobile phases A and B were 2% ACN with 0.1% formic acid, and 98% ACN with 0.1% formic acid, respectively. Trapping and desalting were carried out with solvent A for 15 min at a flow rate of 350 nL/min. Analytical separation was accomplished using 5% B for 5 min at a flow rate of 350 nL/min. A linear gradient of 5–35% of mobile phase B was applied during the next 60 min. Subsequently the gradient was increased to 95% B within 5 min and maintained for the next 12 min. B was then decreased to 5% within 3 min and maintained for 5 additional min. MS analysis was conducted with a TripleTOF 5600 System (AB SCIEX, Concord, ON, Canada) in Information Dependent Mode. Parameter settings were as described by Andrews et al. (46).

### Peptide and Protein Identification

For iTRAQ quantitation, peptides were automatically selected by the Pro Group^TM^ algorithm to calculate the reporter peak area (47). The algorithm uses only ratios that are unique to a protein to avoid calculating artifacts that can occur when peptides common to both proteins are included. Data were automatically corrected for bias to remove variations caused by unequal mixing during sample preparation. Differences in protein abundance in adhesive and non-adhesive piglets were evaluated using a *t* test. Differentially expressed proteins (DEPs) were identified using an FDR-adjusted significance threshold of *P* < 0.05 and fold change (FC) > 1.5. A small number of proteins were excluded from the bioinformatics analysis because they exhibited large variations amongst the four replicates. In these cases, it is possible that significant differences in levels may be the result of detection errors.

### Bioinformatics Analysis

Protein identification and relative iTRAQ quantification were performed with ProteinPilot^TM^ 4.2 (AB SCIEX, USA) in which peptides were identified using the Paragon^TM^ algorithm. Data were further processed using the Pro Group^TM^ algorithm, which performs isoform-specific quantification (47). Peptides were compared to entries in the NCBInr database (69110 sequences; http://www.ncbi.nlm.nih.gov/protein), concatenated with a decoy database containing randomized sequences from the original database. Pathway enrichment analysis for DEPs was conducted using the PANTHER (protein annotation through evolutionary relationship) classification system (http://www.pantherdb.org/) (14). Data were analyzed using a statistical overrepresentation test and statistical enrichment test. The numerical data of our work is the fold-change value for each protein in the differential pairs. DEPs were used as queries in the Search Tool for the Retrieval of Interacting Genes/Proteins (STRING; http://string.embl.de/) to build a functional protein association network (48). BioCircos (49) was used to visualize the genomic location of DEGs.

### Construction of CRISPR/Cas9–sgRNA Expression Vector

Single-guide RNAs (sgRNAs) targeted to exon 1 and 2 of *Sus scrofa* integrin subunit beta 5 (*ITGB5*) were designed using online CRISPR design tools (http://crispr.mit.edu/) (50). Six sgRNAs (Figure 4A, Table 2) were selected for expression vector construction using clustered regularly interspaced short palindromic repeats (CRISPR)/CRISPR–associated protein 9 (Cas9)–sgRNA, based on their predicted scores and lower off-target effects. DNA oligonucleotides corresponding to the sgRNAs were synthesized by Invitrogen (Shanghai, China). Annealed oligonucleotides were inserted into pX330-U6-Chimeric_BB-CBh-hSpCas9 (plasmid 42230, PX330, Addgene, a gift from Feng Zhang, Broad Institute of MIT and Harvard) containing two *BbsI* (R3539S, NEB, Ipswich, MA) restriction enzyme sites, using a published protocol (51). The sgRNA with higher efficiency was used for single clone selection.

**Table 2:** *In vitro* cleavage efficiency of Cas9–sgRNA at target sites.

**Name of sgRNAs**	**Target sequences (5**^′^** to 3**^′^**)**	***In vitro* cleavage activity, %**
ITGB5-g1	GCCCGAAGAGGCAGGCGTAC	0
ITGB5-g2	CGAGCGCGCAGAGCCCGAAG	0
ITGB5-g3	CCGAAGAGGCAGGCGTACAG	0
ITGB5-g4	GCAGGCGTACAGGGGCGCGG	11.8
ITGB5-g5	CAGACAGTCTTCACACGAGG	15.5
ITGB5-g6	AAGCAGACAGTCTTCACACG	10.2

### Vector Plasmid Transfection, DNA Extraction, and T7EN1 Assay

sgRNA cleavage activity was validated by co-transfection of IPEC-J2 cells with the CRISPR/Cas9–sgRNA and pEGFP-C1 plasmids, which included genes encoding puromycin resistance and enhanced green fluorescent protein (eGFP). An empty plasmid was used as a negative control. IPEC-J2 cells were cultured in 6-well plates to 70–80% confluence. Transfection was performed at the ratio of 1 μg : 1 μg : 2.5 μL for the knockout plasmid, pEGFP-C1 plasmids, and Lipofectamine 2000 (11668019, Invitrogen, Waltham, MA), respectively. Forty-eight hours after transfection, viable cells that were positive for green fluorescent protein (GFP) were collected, and genomic DNA was extracted using a University Genomic DNA Kit (CW Biotech, China). The genomic region flanking the target site was PCR amplified using the test primers (Table S4). DSBs (double strand breaks) introduced by CRISPR/Cas9 are primarily repaired by NHEJ (non-homologous end joining), which often generates “indels” around cleaving site. If indels emerged and formed mismatches with wild type DNA, it could be detected via T7EN1 (T7 endonuclease I) assay because T7EN1 enzyme is sensitive to DNA mismatches (52). Also, T7EN1 is the preferred enzyme to scan mutations triggered by CRISPR/Cas9 and evaluate knockout efficiency (53). Purified PCR products were annealed before conducting a T7 endonuclease I (T7EN1)-cleavage assay (M0302L, NEB) (53). Digestion products were analyzed by agarose gel electrophoresis. Band intensities were measured using ImageJ (ImageLab, http://imagej.net). The PCR product enzyme digestion frequency, *fcut*, was determined using the formula (*b* + *c*)/(*a* + *b* + *c*), where *a* is the intensity of the undigested PCR product, and *b* and *c* are the intensities of the cleavage bands. Indel formation was estimated from *fcut* using the binomial probability distribution:

$$\begin{array}{l} \text{indel }\left(\%\right)=[1-\sqrt{(1-f_{cut\;}})]\;\times \;100\% \end{array}$$

### Establishment of Cell Line With *ITGB5* Gene Knockout

CRISPR/Cas9–sgRNA and pEGFP-C1 plasmids were transfected into IPEC-J2 cells using Lipofectamine 2000. Cells transfected with pEGFP-C1 and PX330 but without sgRNA served as a control. Puromycin selection was performed 48 h after transfection and maintained 8–10 days until all control cells died. After selection, cells were counted using a hemocytometer and diluted to a final concentration of 1 cell per 100 μL. Individual cells were then transferred to 96-well plates and cultured for 10–14 days to obtain single-clone colonies. Cells from each colony were collected by trypsinization, and the cell line was gradually expanded by sequential passage through cultures in 24-well plates, 12-well plates, and 6-well plates. Genomic DNA extracted from single clones was used as a PCR template, and the products were inserted into the PMD19-T vector. TA clones were analyzed by sequencing (Invitrogen, Shanghai, China). The workflow is summarized in Figure 4B.

### Cloning the *ITGB5* Into pEGFP-C1

A full-length cDNA encoding the porcine *ITGB5* gene was synthesized by Invitrogen (Shanghai, China). The product was cloned into the pEGFP-N1 vector at the Bgl II and Kpn I sites after restriction enzyme digestion and ligation using T4 DNA ligase (New England BioLabs). The resulting construct, pEGFP-N1-ITGB5, expressed the sense strand of the gene.

### Quantitative RT-PCR Analysis

Total RNA was extracted from cells with TRIzol reagent (Invitrogen, USA) and reverse-transcribed to cDNA. The qRT-PCR reactions were performed with the Bio-Rad CFX96^TM^ Real-Time System (Bio-Rad). The GAPDH gene was served as an internal reference gene, and all reactions were performed in triplicate. Gene expression levels were calculated using the 2^−ΔΔCt^ method.

### Adhesion Assay

Bacterial adhesion to IPEC-J2 cells was evaluated by real-time PCR using procedures described by Candela et al. (54) with slight modification. Briefly, cells (*ITGB5*-knockout, *ITGB5*-overexpression, and control cells) were cultured in 6-well plates until reaching 90% confluence. The cells were washed three times with PBS buffer, and then 1 ml of DMEM/F12 and 30 μL of F4ac ETEC strain 200 [10^8^ CFU/mL, MOI (multiplicity of infection) = 200:1] were added. Cells and bacteria were then co-incubated at 37°C in a 5% CO_2_-95% air atmosphere for 4 h. Unattached bacteria were removed by washing the monolayers four times with sterile PBS. The remaining (attached) bacterial cells were quantified by real time PCR performed with the STa primers listed in Table S4. Serial dilutions of bacteria in PBS (1 × 10^5^-1 × 10^9^ CFU/mL) were also subjected to real time PCR and used as standards.

### GST Pull-Down Assay

The full length of the *FaeG* gene was cloned into pGEX-4T-1 for fusion with a GST tag, and the fragments of *ITGB5* with the transmembrane region eliminated was cloned into pCzn1 for fusion with an N-His tag. Recombinant protein was expressed in the *E.coli* strain Rosetta and purified. The fusion protein of GST-FaeG and GST (control) was then bound to glutathione agarose beads for 4 h at 4°C and then washed. His-ITGB5 was purified and desalinated, and then they were incubated with the glutathione agarose beads bounded with GST-FaeG or GST at 4°C overnight, respectively. Next, the mixture was washed by PBS 3 times, and then the beads-bound proteins were eluted by boiling in PAGE buffer for 30 min. Finally, Western blotting were performed to determine whether FaeG and ITGB5 interact *in vitro*. The blots were incubated overnight with either anti-GST antibody or anti-His antibody, and they were then stained using enhanced chemiluminescence (ECL) (Pierce) regents.

## Data Availability Statement

The mass spectrometry proteomics data have been deposited to the ProteomeXchange Consortium (http://proteomecentral.proteomexchange.org) via the iProX partner repository with the dataset identifier PXD013722.

## Ethics Statement

The animal study was reviewed and approved by Institutional Animal Care and Use Ethics Committee of Shandong Agricultural University.

## Author Contributions

QZ, WW, and YY conceived this study. WW and YL were responsible for animal care, prepared samples, and performed the experiments. QZ, YY, WW, and HT performed the data processing and wrote the manuscript. All authors reviewed and approved the final manuscript.

### Conflict of Interest

The authors declare that the research was conducted in the absence of any commercial or financial relationships that could be construed as a potential conflict of interest.
