# Supplementary material for: ITGB5 Plays a Key Role in Escherichia coli F4ac-Induced Diarrhea in Piglets
Source: Front Immunol. 2019 Dec 11;10:2834. doi: 10.3389/fimmu.2019.02834 (PMC6927286; doi:10.3389/fimmu.2019.02834)

**Figure S1** Protein classification for proteins identified in the iTRAQ experiment.


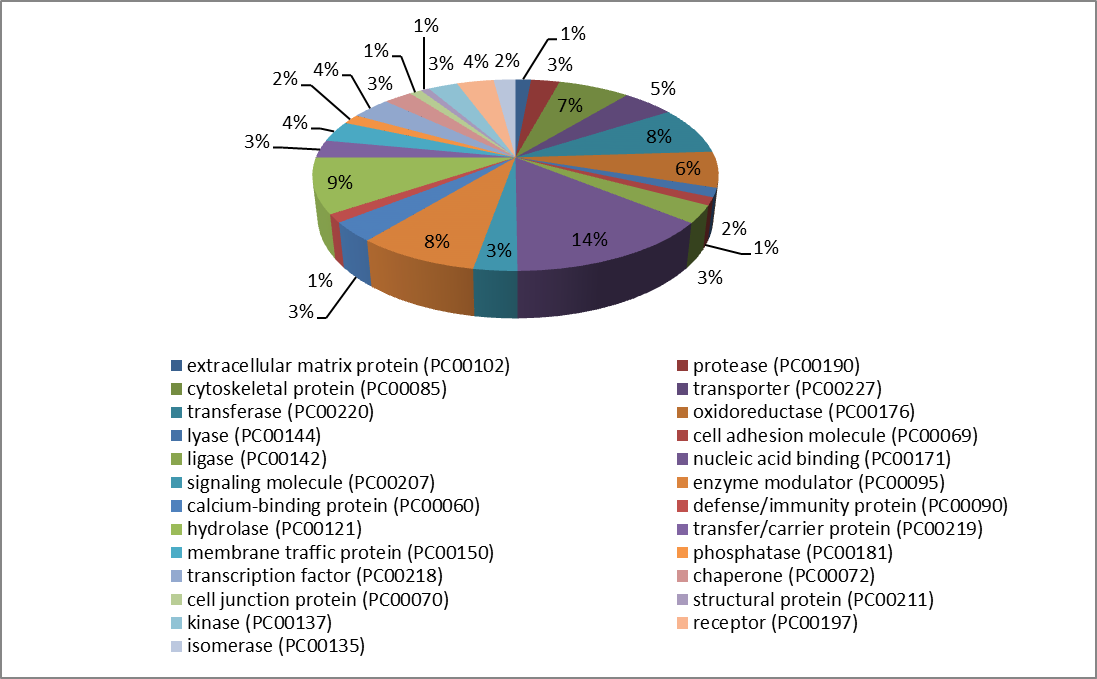


**Figure S2** Volcano plot comparing protein expression in full-sibs.

**113 *vs* 117**


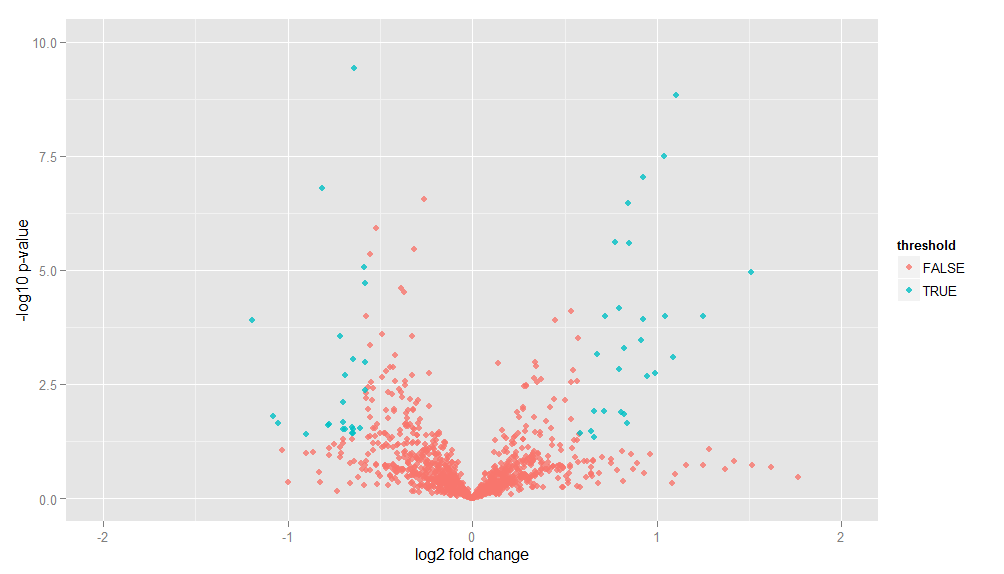


**114 *vs* 118**


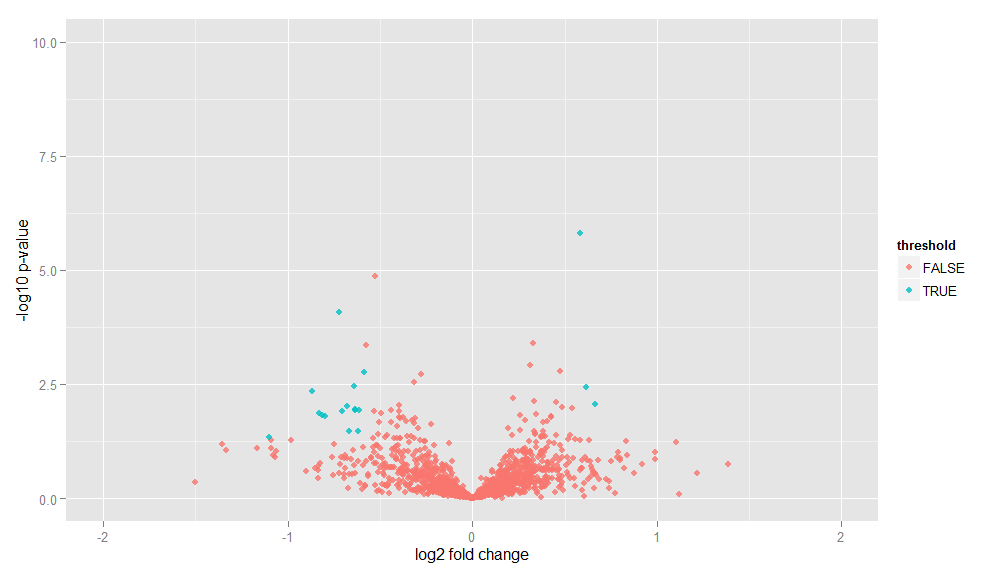


**115 *vs* 119**


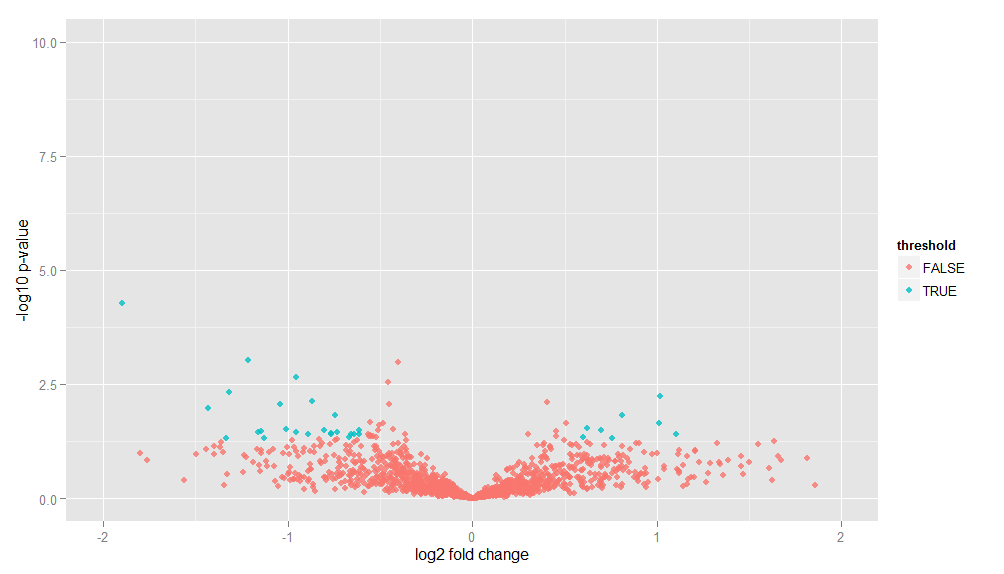


**116 *vs* 121**


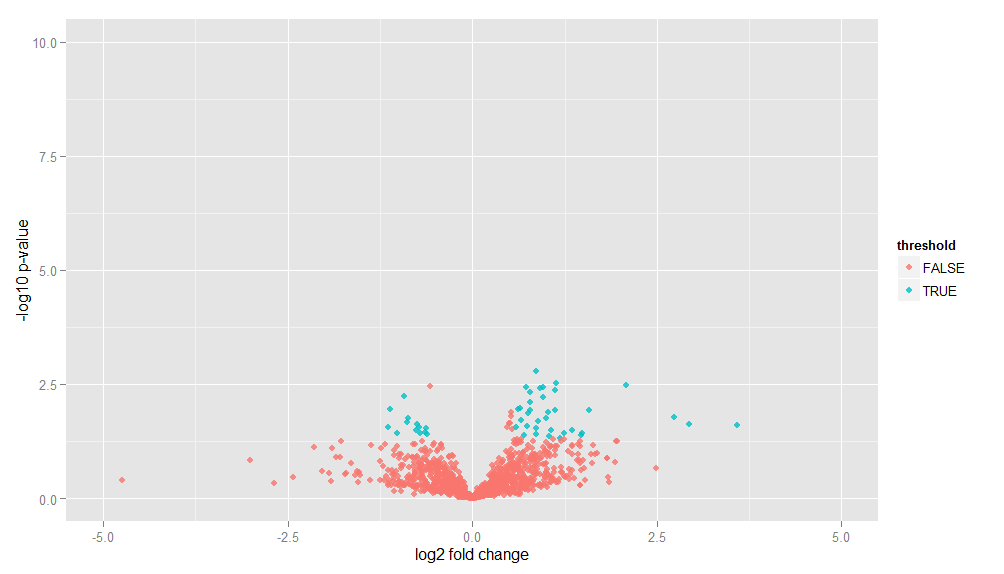

Supplement: Supplementary file 1 [file Data_Sheet_1.docx]
